# Control of Theta Oscillatory Activity Underlying Fear Expression by mGlu_5_ Receptors

**DOI:** 10.3390/cells11223555

**Published:** 2022-11-10

**Authors:** Pawel Matulewicz, Arnau Ramos-Prats, Xavier Gómez-Santacana, Amadeu Llebaria, Francesco Ferraguti

**Affiliations:** 1 Institute of Pharmacology, Medical University of Innsbruck, Peter-Mayr-Str. 1, 6020 Innsbruck, Austria; 2 Department of Animal and Human Physiology, Faculty of Biology, University of Gdansk, Jana Bazynskiego 8, 80-309 Gdansk, Poland; 3 Laboratory of Medicinal Chemistry & Synthesis (MCS), Department of Biological Chemistry, Institute for Advanced Chemistry of Catalonia (IQAC-CSIC), Jordi Girona 18-26, 08034 Barcelona, Spain

**Keywords:** metabotropic glutamate receptors, fear conditioning, theta rhythm, ventral hippocampus, medial prefrontal cortex

## Abstract

Metabotropic glutamate 5 receptors (mGlu_5_) are thought to play an important role in mediating emotional information processing. In particular, negative allosteric modulators (NAMs) of mGlu_5_ have received a lot of attention as potential novel treatments for several neuropsychiatric diseases, including anxiety-related disorders. The aim of this study was to assess the influence of pre- and post-training mGlu_5_ inactivation in cued fear conditioned mice on neuronal oscillatory activity during fear retrieval. For this study we used the recently developed mGlu_5_ NAM Alloswicth-1 administered systemically. Injection of Alloswicth-1 before, but not after, fear conditioning resulted in a significant decrease in freezing upon fear retrieval. Mice injected with Alloswicth-1 pre-training were also implanted with recording microelectrodes into both the medial prefrontal cortex (mPFC) and ventral hippocampus (vHPC). The recordings revealed a reduction in theta rhythmic activity (4–12 Hz) in both the mPFC and vHPC during fear retrieval. These results indicate that inhibition of mGlu_5_ signaling alters local oscillatory activity in principal components of the fear brain network underlying a reduced response to a predicted threat.

## 1. Introduction

Group I metabotropic glutamate receptors (mGlus), namely mGlu_1_ and mGlu_5_, have recently been suggested to contribute to affective behavior [1,2,3,4]. They display a largely complementary distribution [5] with mGlu_5_ most abundantly expressed in telencephalic regions, such as the hippocampus, neocortex and striatum [6,7]. Numerous studies have implicated mGlu_5_ in neuropsychiatric disorders including autism, schizophrenia, depression and anxiety disorders [8,9,10,11,12]. Several imaging studies in humans have shown a close relationship between mGlu_5_ levels and symptom severity in patients suffering from post-traumatic stress disorder and major depression [13,14]. In preclinical studies, antagonists and negative allosteric modulators (NAMs) of mGlu_5_ were consistently found to exert anxiolytic-like effects in a broad variety of tests including conflict tasks, such as the elevated plus maze (EPM), and the light dark box, as well as in fear conditioning [9,15,16,17,18,19,20,21,22,23,24,25]. Impaired acquisition of fear responses has been described in mice carrying the deletion of the mGlu_5_ gene (*Grm5*-KO) [26,27]. Moreover, the administration of mGlu_5_ antagonists or NAMs before fear conditioning hampered the conditioned threat response, pointing towards a critical role of mGlu_5_ signaling during CS–US associations [20,21,22,23,28]. However, the mechanisms by which mGlu_5_ NAMs affect the principal components of the fear brain network remained unaddressed so far.

Considerable evidence supports a link between emotional states and oscillatory activity in the brain. Previous studies have shown that the initiation and expression of defensive behavior in rodents, such as freezing, is characterized by enhanced rhythmicity in the theta range in the medial prefrontal cortex (mPFC), ventral hippocampus (vHPC) and basolateral amygdala (BLA) [29,30,31,32,33,34,35,36,37,38,39]. Theta rhythm is a highly synchronous pattern of neuronal oscillations, with high voltage and frequency range of 4–12 Hz [29,30,40,41,42,43]. Theta oscillations reflect synchronized neural firing and are believed to facilitate long-range communication between brain areas involved in the processing and expression of anxiety and fear [30,31,34,36,44,45]. However, no studies have addressed whether the potent anxiolytic-like and fear-reducing action of mGlu_5_ NAMs is accompanied or mediated by changes in theta oscillatory activity in the fear network during aversive state processing.

In this study, we have characterized the effects of the systemic administration during fear conditioning of the novel mGlu_5_ NAM Alloswitch-1 [46] on the oscillatory activity in principal hubs of the fear and anxiety brain network during the retrieval of fear memory in male mice. Our data show that Alloswitch-1, when given pre- but not post-training, alters theta (4–12 Hz) activity in the mPFC and vHPC underlying a reduced response to a predicted threat.

## 2. Materials and Methods

### 2.1. Implantations

Male mice (C57BL/6j, 8–12 week-old) were stereotactically (Kopf Instruments, Tujunga, CA, USA) implanted under sevoflurane (Sevorane, AbbVie GmbH, Vienna, Austria) anesthesia combined with ketamine/xylazine (i.p.), with recording electrodes made of twisted 76,2 µm teflon coated, stainless steel wires (Science Products, Hofheim, Germany) into the mPFC (at a 3° angle, AP: +1.8, L: +0.5, D: −1.7 mm) and vHPC (AP: −3.2, L: +3.3, D: −2.8 mm). The stereotaxic coordinates were based on the Franklin and Paxinos Mouse Brain Atlas [47]. A silver wire (Science Products, Hofheim, Germany) connected to a screw mounted posteriorly to the bregma was used as ground/reference electrode. Two small screws were also mounted to the skull for additional support. All electrodes were connected to a 10-pin PCB connector and cemented to the skull with dental acrylic (Paladur, Heraeus Kulzer GmbH, Hanau, Germany). During the surgery, ophthalmic ointment and an analgesic-meloxicam (Metacam, Boehringer Ingelheim; 0.01 mg/kg subcutaneously) were applied. After 7–10 days of recovery, animals were habituated for nearly a week to the experimenter (handling) and to the recording setup (2–3 sessions, lasting approximately 10 min). In one set of experiments (1st Set), animals were blindly assigned to two groups: one (*n* = 14) receiving Alloswitch-1 (10 mg/kg) and the other (control; *n* = 13) receiving the drug vehicle (Saline + 5% DMSO + 1% TWIN80), injected i.p. in a volume of 0.20–0.23 mL (depending on the animal’s body weight), 15 min before the fear acquisition session. In an independent 2nd set of experiments, unimplanted, naïve mice were injected with either Alloswitch-1 (10 mg/kg, *n* = 6) or drug vehicle (Saline + 5% DMSO + 1% TWIN80, *n* = 6), immediately after the fear acquisition session.

Alloswitch-1 is an azobenzene derivative of VU0415374 (MW 381), which has been shown to display good brain penetrance at 10 mg/kg when administered systemically [48] and in vivo actions following intracerebral administration at doses similar to those used for MPEP [49]. We and others have previously shown behavioral effects of conventional mGlu_5_ NAMs at doses between 3 and 30 mg/kg when administered systemically, within a time range of 15 min to 1 h [25,50,51,52]. Therefore, based on the structural similarity to VU0415374, known potency in vivo of other brain penetrant mGlu_5_ NAMs and own experience, we considered the i.p. administration of 10 mg/kg given 15 min before behavioral testing as a suitable experimental condition for this study.

All procedures involving animals were approved by the Austrian Animal Experimentation Ethics Board and were performed in compliance with the European Convention for the Protection of Vertebrate Animals used for Experimental and Other Scientific Purposes (ETS no. 123).

### 2.2. Fear Conditioning and Retrieval Protocol

Fear conditioning and retrieval were performed in a 27 cm × 27 cm × 40 cm chamber with transparent walls and a metal grid on the floor for foot-shock delivery (Ugo Basile, Comerio, Italy). Mice were placed in the conditioning chamber for a 60 sec baseline period and then subjected five times to a 15 sec-long 60 dB white noise conditioned stimulus (CS) followed by a foot-shock (0.5 mA) unconditioned stimulus (US) lasting 1 sec and with a 1 min inter-trial interval between each CS-US presentation. Freezing (%) during the CS presentations was taken as a measure of fear conditioning/learning. Twenty-four hours later, animals were exposed to the fear retrieval session in the same context (recording chamber) and were presented 5 times with the CS (with 1 min interval) without the reinforcing US. The same context was used in order to induce stronger theta activity. The chamber was cleaned with 70% EtOH between subjects. Mice were tracked using contour tracking and center of mass via ANY-maze (Stoelting Europe, Dublin, Ireland), using a video camera mounted on top of the fear conditioning/retrieval chamber. The automatic freezing assessment was inaccurate due to cable movements; therefore, freezing was manually scored by a trained experimenter blind to the treatment. The freezing score is expressed as percentage of immobility/freezing time during the CS presentations.

### 2.3. LFP Signal Acquisition and Analysis

During each fear retrieval session, local field potential (LFP) signals from mPFC and vHPC were recorded. LFP signals were recorded on an EXT-9 recording system using a headstage-commutator assembly (NPI electronic GmbH, Tamm, Germany) allowing animals to move freely inside the fear conditioning chamber. The raw signal was amplified ×1000, filtered from 0.1 to 1000 Hz, digitized at 1 kHz (Power 1401, CED, Cambridge, UK) and stored on a PC by means of the Spike 2 (version 8.08) software (CED, Cambridge, UK). Only animals that were positively verified regarding the LFP signal quality (appropriate signal amplitude, no movement artefacts) during the recording session were used for further off-line signal analysis. Artefacts-free 15 sec LFP signal epochs from the CS presentation periods during the fear retrieval session were taken and analyzed using the MATLAB software (version 2020b, Mathworks, CA, USA). The spectral analysis of the recorded LFP signal was calculated using the Welch’s power spectral density (PSD) estimate method (pwelch.m MATLAB function), computing 1 s signal segments with a 1000 Hz sampling rate and a 50% overlap. Afterwards, data points were transformed into Z-scores (range 1–48 Hz). The correlation between the amount of freezing (%) and the mean signal power (expressed as Z-score) of the dominant frequency (frequency with the highest Z-scored signal power) within the theta band (4–12 Hz) during CS presentations of the fear retrieval session was analyzed using Pearson’s r.

### 2.4. Histology

At the end of the behavioral experiments, mice were perfused with a fixative to confirm the correct location of the LFP recording electrodes in the brain. Mice were subjected to a non-recovery anesthesia with thiopental sodium (150 mg/kg, i.p.) and perfused transcardially at first with saline (0.9% NaCl), followed by a fixative made of 4% paraformaldehyde in 0.1 M phosphate-buffer (PB), pH 7.2–7.4, for 12 min. Immediately after the perfusion, brains were removed from the skull and stored in 4% PFA solution at 6 °C until further use. Brains were cut with a vibratome (Leica VT1000S; Leica Microsystems, Vienna, Austria) into 50 μm thick coronal slices that were collected in six wells Petri-dishes filled with 0.1 M PB + 0.05% NaN_3_. Sections containing the mPFC and vHPC were mounted on gelatin-coated glass slides (Thermo Scientific) and stained with Cresyl Violet (Nissl staining). Implantation sites (Supplementary Figure S1) were assessed using a Zeiss AxioImager Z1 microscope (Carl Zeiss Microimaging GmbH, Göttingen, Germany) by an experimenter blinded to the treatment condition.

### 2.5. Statistics

Sample size was predetermined based on published studies, experimental pilots, and in-house expertise. All statistical analyses were performed on GraphPad Prism (ver. 9.0.1). Following normality checks, all data were analyzed using unpaired two-tailed *t*-test or two-way RM ANOVA with Bonferroni’s multiple comparison test (following significant ANOVA). In each case, * *p* < 0.05 was considered as the significance threshold.

## 3. Results

### 3.1. Inhibition of mGlu_5_ during Fear Conditioning Reduces CS-US Association

**Figure 1:**
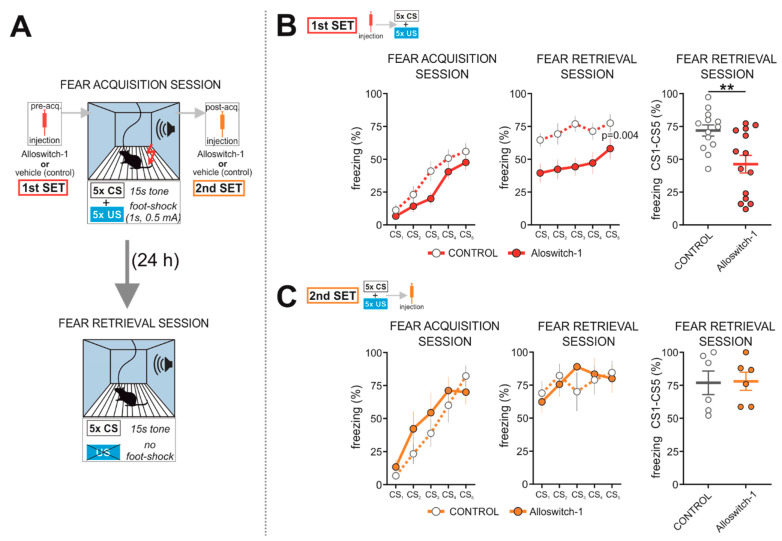
(**A**) Experimental design. (**B**) The first set (SET 1) of animals was i.p. injected with Alloswitch-1 (*n* = 14) or vehicle (*n* = 13) before the fear acquisition session. (**C**) A second set of animals (SET 2), not implanted with microelectrodes, received the i.p. injection of Alloswitch-1 (*n* = 6) or vehicle (*n* = 6) right after the end of the fear acquisition session. (**B**,**C**) Freezing levels during consecutive CS-US presentations in the course of fear acquisition (left panels) or CS presentations during fear retrieval (central panels). Data were analyzed by two-way repeated measures ANOVA. Right panels depict the mean % freezing across all 5 CS presentations during the fear retrieval session. Data are presented as mean ± SEM, including individual values, and analyzed by the unpaired two-tailed *t*-test, ** *p* ≤ 0.01.

Mice were subjected to cued fear conditioning, in which they were exposed five times to a neutral auditory CS terminating with a mild foot shock (US), followed by fear retrieval 24 h later, where only the CS was presented in the same context (Figure 1A). Alloswitch-1 or vehicle (control group) was injected i.p. before (Figure 1B) or right after the fear acquisition session (Figure 1C). Mice treated before fear conditioning were also implanted with recording electrodes in the vHPC and mPFC (Supplementary Figure S1). They showed a progressive increase in freezing upon subsequent CS presentations, demonstrating a successful acquisition of a conditioned response, whereas Alloswitch-1 did not significantly influence the amount of freezing compared to the control group (Figure 1B; left panel: 2-way RM ANOVA: drug F(1,25) = 2.537, *p* = 0.1237; time F(4,100) = 55.45, *p* < 0.0001; drug × time F(4,100) = 1.658, *p* = 0.1659). However, we could observe a tendency towards a reduced or delayed conditioned response in the Alloswitch-1 injected mice, consistent with previous studies [23]. During the fear retrieval session, 24 h later, Alloswitch-1-treated mice exhibited a profound reduction in freezing in comparison to control animals (Figure 1B; central panel: 2-way RM ANOVA: drug F(1,25) = 10.15, *p* = 0.0038; time F(4,100) = 2.686, *p* = 0.0356; drug × time F(4,100) = 0.4520, *p* = 0.7707. right panel: two-tailed *t*-test: t(25) = 3.187, *p* = 0.004).

Mice that received Alloswitch-1 right after fear conditioning showed no differences in the amount of freezing either in the fear acquisition (Figure 1C; left panel: 2-way RM ANOVA: drug F(1,10) = 0.4765, *p* = 0.5057; time F(4,40) = 29.89, *p* < 0.0001; drug × time F (4,40) = 1.614, *p* = 0.1897) or in the fear retrieval session (Figure 1C; central panel: 2-way RM ANOVA: drug F(1,10) = 0.00971, *p* = 0.9234; time F(4,40) = 1.916, *p* = 0.1265; drug × time F(4,40) = 1.249, *p* = 0.3059. right panel: two-tailed *t*-test: t(10) = 0.09856, *p* = 0.9234).

Overall, these results suggest that mGlu_5_ inhibition during fear conditioning reduces the CS-US association strength, whereas post-conditioning inhibition has no effects on fear memory, indicating that mGlu_5_ signaling is not critical for the consolidation of conditioned fear, fully consistent with previous studies using other mGlu_5_ NAMs [21,23,53].

### 3.2. Analysis of mPFC and vHPC Local Neuronal Oscillatory Activity during Fear Retrieval

#### 3.2.1. Signal Power Spectrum

**Figure 2:**
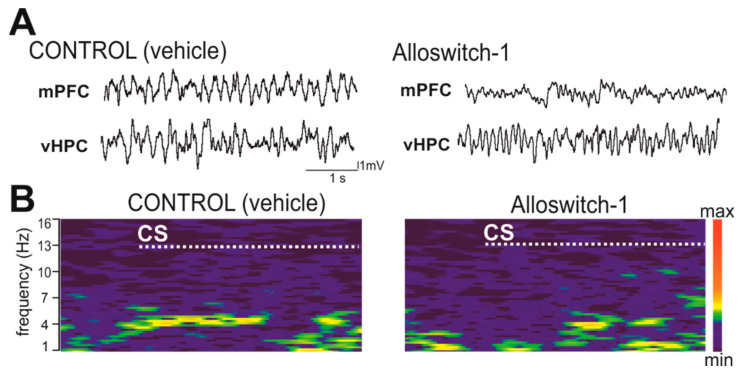
(**A**) Representative fragments of recorded LFPs from the mPFC and vHPC during a CS presentation in the course of the fear retrieval session. (**B**) Examples of LFPs expressed as sonograms recorded from the mPFC of a vehicle- (control) or Alloswitch-1 injected animal (prior to the fear acquisition session) at the time of a CS presentation (CS 3). The dotted line defines the time of the CS presentation. A distinct increase in the signal power at 4–6 Hz within the theta frequency band (4–12 Hz) can be observed concomitant with the beginning of the CS in the control animal, but not in the Alloswitch-1 injected mouse, which, however, showed some bouts of theta activity demonstrating an intact ability to induce it.

**Figure 3:**
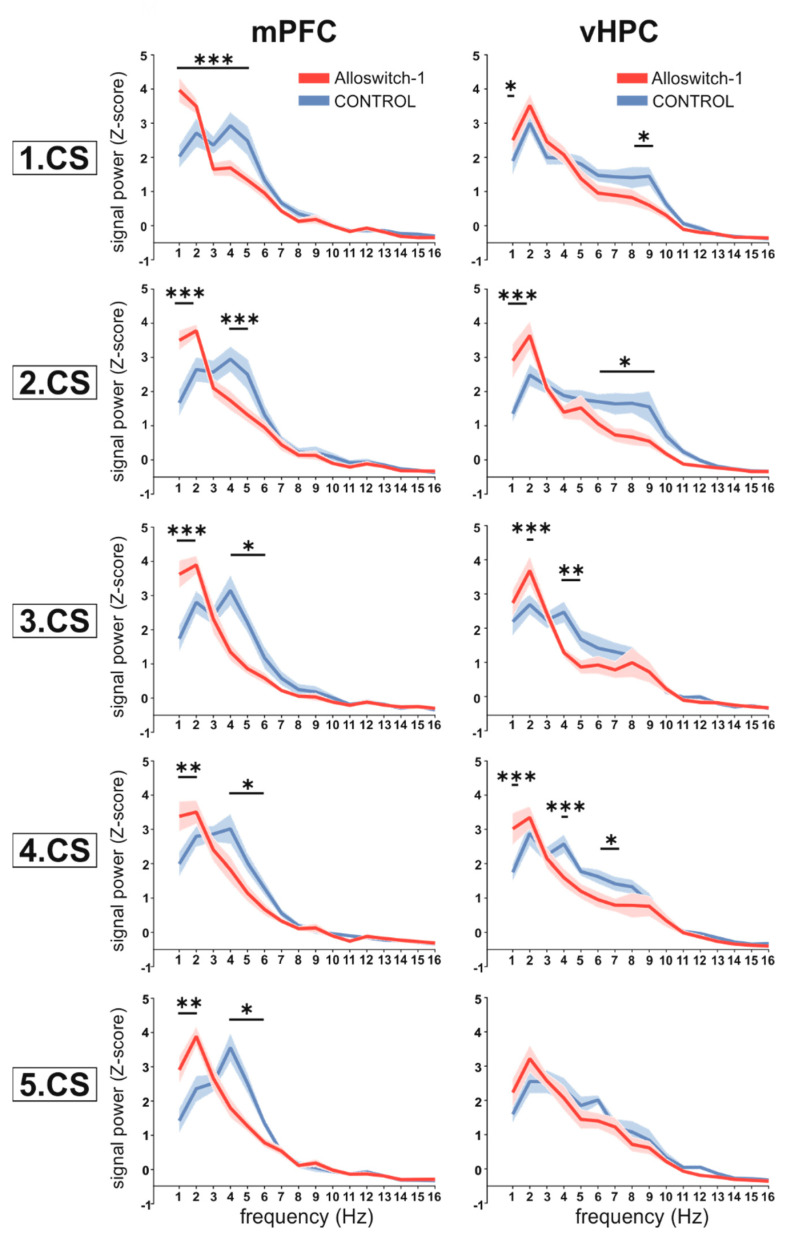
Power spectra (Z-scored) of the LFP signal recorded from the mPFC (*n* = 10) or vHPC (*n* = 10) during consecutive CS presentations (CS1-CS5) in the course of the fear retrieval session in animals that were injected with Alloswitch-1 or vehicle before fear conditioning. Data are shown as mean ± SEM and were analyzed by two-way RM ANOVA followed by Bonferroni’s multiple comparison test to compare Z-scores between animals treated with Alloswitch-1 and controls, * *p* ≤ 0.05, ** *p* ≤ 0.01, *** *p* ≤ 0.001. In case of significant differences within multiple subsequent frequencies (1 Hz bins), only the lowest level of significance is shown.

Next, we sought to explore whether the reduced freezing observed upon fear retrieval in mice, that received Alloswitch-1 before fear acquisition, was accompanied by changes in neuronal oscillatory activity in the mPFC and vHPC (Figure 2). The analysis of LFP signals recorded during the CS presentations in the fear retrieval session revealed large differences in the signal power spectra between mice injected with Alloswitch-1 or vehicle (Figure 3).

In particular, we observed a marked reduction in the theta frequency band (4–12 Hz) of the spectral content in the mPFC (Figure 3, left panels) in Alloswitch-1-treated animals (CS1: 2-way RM ANOVA: drug × frequency F(46,828) = 6.906, *p* < 0.0001; frequency F(46,828) = 135.7, *p* ≤ 0.0001; drug F(1,18) = 9.499, *p* = 0.0064; CS2: 2-way RM ANOVA: drug × frequency F(46,828) = 6.109, *p* < 0.0001; frequency F(46,828) = 120.1, *p* ≤ 0.0001; drug F(1,18) = 8.820, *p* = 0.0082; CS3 2-way RM ANOVA: drug × frequency F(46,828) = 7.015, *p* < 0.0001; frequency F(46,828) = 101.3, *p* ≤ 0.0001; drug F(1,18) = 10.35, *p* = 0.0048; CS4: 2-way RM ANOVA: drug × frequency F(46,828) = 7.708, *p* < 0.0001; frequency F(46,828) = 126.2, *p* ≤ 0.0001; drug F(1,18) = 3.247, *p* = 0.0883; CS5: 2-way RM ANOVA: drug × frequency F(46,828) = 3.845, *p* < 0.0001; frequency F(46,828) = 113.3, *p* ≤ 0.0001; drug F(1,18) = 5.866, *p* = 0.0262). Similarly, analysis of the LFP signals recorded from the vHPC (Figure 3, right panels) also revealed significant differences in the average power spectra of the LFP signals between mice injected with Alloswitch-1 and vehicle before the fear acquisition session (CS1: 2-way RM ANOVA: drug × frequency F(46,828) = 2.075, *p* < 0.0001; frequency F(46,828) = 113.8, *p* ≤ 0.0001; drug F(1,18) = 4.691, *p* = 0.0440); CS2: 2-way RM ANOVA: drug × frequency F(46,828) = 4.419, *p* < 0.0001; frequency F(46,828) = 82.65, *p* ≤ 0.0001; drug F(1,18) = 8.804, *p* = 0.0083; CS3: 2-way RM ANOVA: drug × frequency F(46,828) = 2.284, *p* < 0.0001; frequency F(46,828) = 84.80, *p* ≤ 0.0001; drug F(1,18) = 1.784, *p* = 0.1983; during CS4: 2-way RM ANOVA: drug × frequency F(46,828) = 2.890, *p* < 0.0001; frequency F(46,828) = 101.9, *p* ≤ 0.0001; drug F(1,18) = 6.919, *p* = 0.0170; CS5: 2-way RM ANOVA: drug × frequency F(46,828) = 1.056, *p* = 0.3749; frequency F(46,828) = 93.78, *p* ≤ 0.0001; drug F(1,18) = 3.795, *p* = 0.999).

Taken together, these results show that upon fear retrieval, the CS presentations were accompanied by the induction of theta oscillations with a distinct peak in the 4–6 Hz frequency range in both the mPFC and vHPC, although in the latter one, the average power of the signal was weaker. Treatment with the mGlu_5_ NAM Alloswitch-1 during fear conditioning produced a shift in the peak power towards lower frequencies in both brain structures (Figure 3).

#### 3.2.2. Alloswitch-1-Treatment Influences the Power of the 4–12 Hz Frequency Band

**Figure 4:**
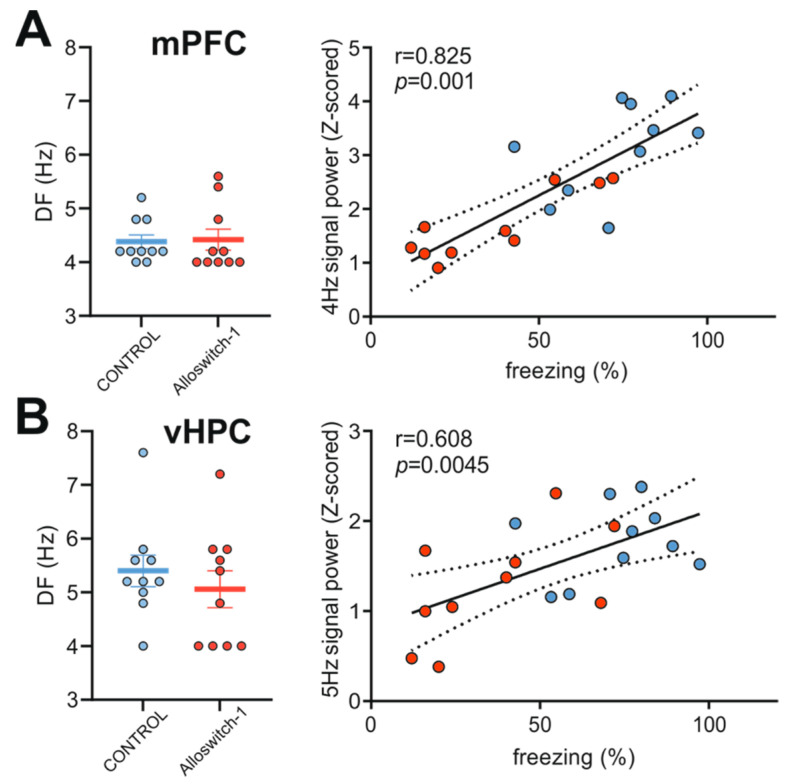
(**A**) Mean dominant frequency (DF) of the signal in the theta frequency band (4–12 Hz) recorded from mPFC (left panel) and (**B**) vHPC (left panel). A strong positive correlation was observed between the power of the LFP signal at the DF in the mPFC (**A**, right panel) and vHPC (**B**, right panel) and freezing during CS presentations. Data are shown as mean ± SEM.

Since low theta oscillations in the mPFC and vHPC have been previously linked to negatively valenced emotional states [29,30,40,42,43,46], we next sought to characterize whether the reduced expression of fear during fear retrieval in mice injected with Alloswitch-1 before fear acquisition coincide with a shift in the dominant frequency (DF) of theta oscillations in the mPFC and vHPC, and found no significant differences between Alloswitch-1-injected and control animals, the mean being DF ≈ 4 Hz in the mPFC (Figure 4A, left panel: two-tailed *t*-test: t(18) = 0.1705, *p* = 0.8665) and ≈5 Hz in the vHPC (Figure 4B, left panel: two-tailed *t*-test: t(18) = 0.735, *p* = 0.4609).

Since 4–6 Hz oscillations in the mPFC have been recently shown to be mechanistically different from theta oscillations and to be highly predictive of freezing behavior [54], we next sought to elucidate whether this behavioral expression within CS presentations correlated with the LFP signal power in the mPFC and vHPC at the DF (4 Hz and 5 Hz respectively). Indeed, a robust positive correlation was observed between the amount of freezing and the signal power of the DF in both the mPFC (Figure 4A, right panel: Pearson’s r = 0.825, *p* < 0.0001) and the vHPC (Figure 4B, right panel: Pearson’s r = 0.6079, *p* = 0.0045).

**Figure 5:**
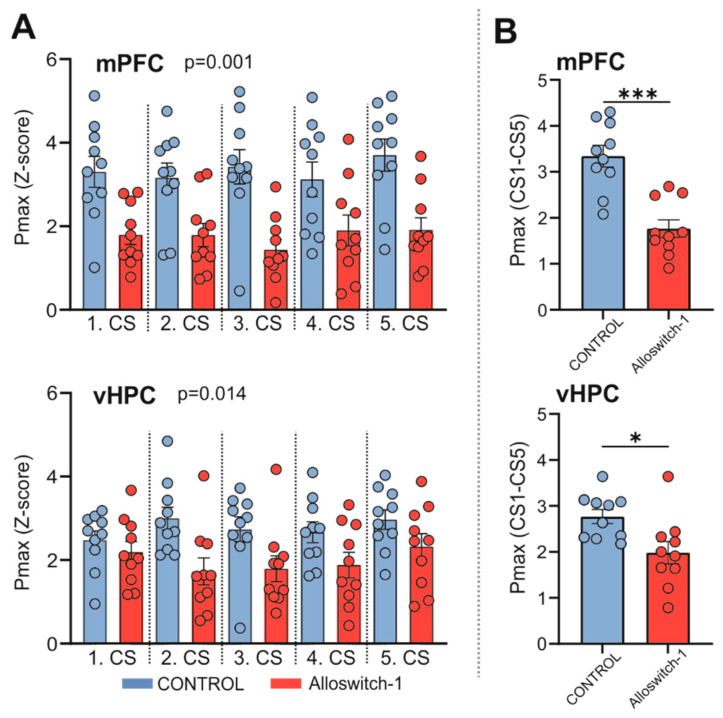
(**A**) Peak power (Pmax, maximum Z-scored signal value) of the LFP signal in the theta frequency band (4–12 Hz) recorded from mPFC (*n* = 10) or vHPC (*n* = 10) during consecutive CS presentations (CS1-CS5). Two-way RM ANOVA (for mPFC drug effect *p* = 0.001, for vHPC *p* = 0.014). (**B**) Mean Pmax value from all CS presentations. Unpaired two-tailed *t*-test, * *p* ≤ 0.05, *** *p* ≤ 0.001. Data are shown as mean ± SEM.

Further analysis of the signal power in the theta frequency range (4–12 Hz) revealed a pronounced decrease in the peak power (Pmax) signal in Alloswitch-1-injected mice in comparison to controls during consecutive CS stimuli in the mPFC (Figure 5A, one-way ANOVA: F(9,90) = 6.292, *p* < 0.0001) and vHPC (Figure 5A, one-way ANOVA: F(9,90) = 2.847, *p* = 0.0054), as well as in the Pmax value expressed as an average of all CS presentations in the mPFC (Figure 5B, two-tailed *t*-test: t(18) = 5.192, *p* < 0.0001) and vHPC (Figure 5B, two-tailed *t*-test: t(18) = 2.724, *p* = 0.0139).

Altogether these findings suggest that mGlu_5_ inhibition results in a decrease in the Pmax, rather than in a shift of the DF within the theta range in both the mPFC and vHPC, which parallels a reduced conditioned response upon fear retrieval.

## 4. Discussion

A large body of evidence shows that inhibition of mGlu_5_ signaling during associative fear learning disrupts conditioned threat responses. This was shown using both genetic and pharmacological approaches, as well as a variety of tests including contextual and cued fear conditioning [21,23,55] and fear-potentiated startle [20,28,56,57,58]. The effect of the reduction in mGlu_5_ activity was mostly observed after conditioning upon fear memory retrieval, with the exception of *Grm5*-KO or the use of high doses of mGlu_5_ antagonists, which attenuated also the acquisition of conditioned fear [22,23,26,27]. Our findings using a different mGlu_5_ NAM, namely, Alloswitch-1 [46], that was never tested in these paradigms before, are fully in line with these studies and further corroborate the view that reduction in mGlu_5_ signaling affects the association between neutral and negatively valenced stimuli. We also observed that pharmacological blockade of mGlu_5_ immediately after fear conditioning, hence, once the stimulus-shock association has already occurred, did not disrupt fear memory consolidation. This is also fully consistent with previous studies showing no effects of mGlu_5_ inactivation following fear conditioning [21,23,53]. We, thus, specifically characterized LFP brain oscillatory activity only in mice that received Alloswitch-1 before the fear acquisition session.

The main finding of our study shows that blockade of mGlu_5_ during fear conditioning resulted in a marked reduction in rhythmic theta-range oscillatory activity (4–12 Hz) during CS presentations upon fear retrieval in both the mPFC and the vHPC. Robust theta activity is known to be associated with high fear states and an enhanced theta synchrony among amygdala–hippocampal–prefrontal cortical circuits was observed during retrieval of conditioned fear [29,30,31,32,36,45,59,60]. Interestingly, activation of mGlu_5_ in the CA3 network in slices was shown to evoke theta frequency oscillations [61], whereas mGlu_5_ inhibition suppressed hippocampal theta activity in dentate gyrus induced by high-frequency tetanization (200 Hz) of the medial perforant path [62]. These data support a direct involvement of mGlu_5_ in the process of generation of neuronal synchronous activity in the theta range.

Arousal states and expression of fear-induced behaviors, such as freezing, are preferentially characterized by a theta oscillation with a frequency range between 4 and 8 Hz, known as type 2 or low theta [37,63,64,65]. Recent studies have also described that following fear conditioning freezing is characterized by a specific oscillatory activity at 4 Hz in the mPFC [54,66], which, in turn, is orchestrated by a 4 Hz breathing frequency [67,68]. Similarly, our study shows that in fear retrieval, during CS presentations, the Pmax was between 4 and 8 Hz and revealed that the administration of Alloswitch-1 before fear conditioning predominantly affected power in this theta frequency range. Moreover, we confirmed that the dominant frequency in the mPFC was at 4 Hz, and that it was highly correlated with freezing, regardless of whether the mice were treated with vehicle or Alloswitch-1. Thus, in mice treated with Alloswitch-1, although we observed reduced signal power throughout type 2 theta, we cannot exclude that the main change affected the 4 Hz respiration-coupled oscillations.

It is worth noting that in the vHPC, we observed a marked reduction in signal power in mice injected with Alloswitch-1 in the high values of type 2 theta (6–10 Hz) during the CS presentations in fear retrieval. This in part overlaps with the 8–12 Hz frequency range also referred to as alpha band. Interestingly, higher values of type 2 theta in the vHPC have been linked to immobility and emotional states such as anxiety and innate fear [69], consistent with our observations.

Memories are thought to be formed and stored by long-term changes in the strength of synaptic connections, a process known as synaptic plasticity [70]. Long-term increases or decreases of synaptic strength have been termed long-term potentiation (LTP) and long-term depression (LTD), respectively [71,72,73]. Learning a CS–US association likely requires the induction of LTP at pathways relaying associative cues [74]. These forms of synaptic plasticity appear to be highly dependent on mGlu_5_ [75,76,77,78,79,80,81,82]. Therefore, the pharmacological inhibition of mGlu_5_ may influence emotional learning and memory retrieval via complex mechanisms occurring at multiple levels, which involve both the induction and maintenance of synaptic plasticity as well as synaptic excitability and synchrony in network activity.

Anxiety disorders in humans are more prevalent in females than males [83] and sex-specific mechanisms are likely to underlie this difference. The effects of mGlu_5_ modulation on fear learning, however, including our study, have been investigated only in male mice, so far. Emerging evidence indicates that the estrous cycle in female rodents exerts a strong influence on fear conditioned responses (e.g., [84,85,86]). Group I mGlus were shown to interact with estrogen receptors (ERs) in females but not in males [87,88] and this interaction was found to produce sex-dependent responses on conflict-based anxiety-like behavior [89,90]. This highlights the likelihood of a complex interaction between mGlu_5_ and ERs during the estrous cycle and in turn an influence on learned fear. Future research should explore in female mice how mGlu_5_ pharmacological inhibition affects fear learning and brain oscillatory activity.

In our study, we used Alloswitch-1, a recently developed and photoswitchable mGlu_5_ NAM [46]. Alloswitch-1 is active as an mGlu_5_ NAM under dark conditions, while under violet light illumination (380–390 nm) its azobenzene group photoisomerizes from *trans* to *cis* configuration, losing its NAM activity; it can, however, quickly re-gain activity under green light (490–500 nm) illumination through a back-photisomerisation to the active *trans* configuration [46]. Alloswitch-1 binds to an allosteric pocket in a similar fashion to other mGlu_5_ NAMs [91,92]. Intra-amygdala injection of Alloswitch-1 in a mouse model of inflammatory pain rapidly and reversibly improved the mechanical pain hypersensitivity, with an efficacy similar to other mGlu_5_ NAMs, such as 2-Methyl-6-(phenylethynyl)pyridine (MPEP) [91]. This compound was also shown to reversibly modulate the behavior of freely moving *Xenopus tropicalis* tadpoles and zebrafish larvae, suggesting that it crosses membranes [46,93]. So far, however, it is still unclear whether Alloswitch-1 can cross the blood–brain barrier. Our work shows, for the first time, a behavioral effect mediated by Alloswitch-1 when administered systemically in mice, that closely reproduces what observed with 3-((2-Methyl-4-thiazolyl)ethynyl)pyridine (MTEP) in a highly similar behavioral paradigm [23]. In addition, given that Alloswitch-1 is a derivative of VU0415374, and that the latter compound was shown to display good brain penetrance at 10 mg/kg when administered systemically [48], it is plausible that Alloswitch-1 also penetrates and acts in the brain. However, we cannot exclude that the effects that we have observed may be mediated entirely or in part through peripheral mechanisms, also in view of the fact that mGlu_5_ were reported to be expressed in peripheral vagal afferents [94]. Similarly, the assumptions about the mechanisms underlying the influence on Pavlovian fear conditioning mediated by other mGlu_5_ NAMs, resulted from studies that also applied them systemically, may have to be adjusted to keep into consideration this possibility. Future studies taking advantage of the photoswitchable properties of Alloswitch-1 will allow to address this issue through the implantation of optic fibers in specific brain areas, e.g., the vHPC, mPFC or BLA, and the local light inactivation of the drug, after its systemic administration.

In conclusion, our study shows that the pre-training, but not post-training, systemic pharmacological blockade of mGlu_5_ leads to the reduction of type 2 theta rhythms during fear retrieval in the vHPC and mPFC, two of the main hubs of the fear network in the brain, which strongly correlated with reduced expression or recall of fear memories. This emphasizes the contribution of mGlu_5_ activation to associative fear learning and the generation of rhythmic activity in different brain areas related to the emotional state of the animal. Our work informs about new mechanisms by which mGlu_5_ regulate emotional behavior and may participate in anxiety and stress-related disorders.

## Data Availability

The data presented in this study are available on request from the corresponding author.

## Supplementary Materials

The following supporting information can be downloaded at: https://www.mdpi.com/article/10.3390/cells11223555/s1, Figure S1: Histological verification of LFP recording electrode implantation sites in the mPFC and vHPC.
