# Supplementary material for: Control of Theta Oscillatory Activity Underlying Fear Expression by mGlu5 Receptors
Source: Cells. 2022 Nov 10;11(22):3555. doi: 10.3390/cells11223555 (PMC9688906; doi:10.3390/cells11223555)
Supplement: Supplementary file 1 [file cells-11-03555-s001.zip › cells-1881745-supplementary.pdf]

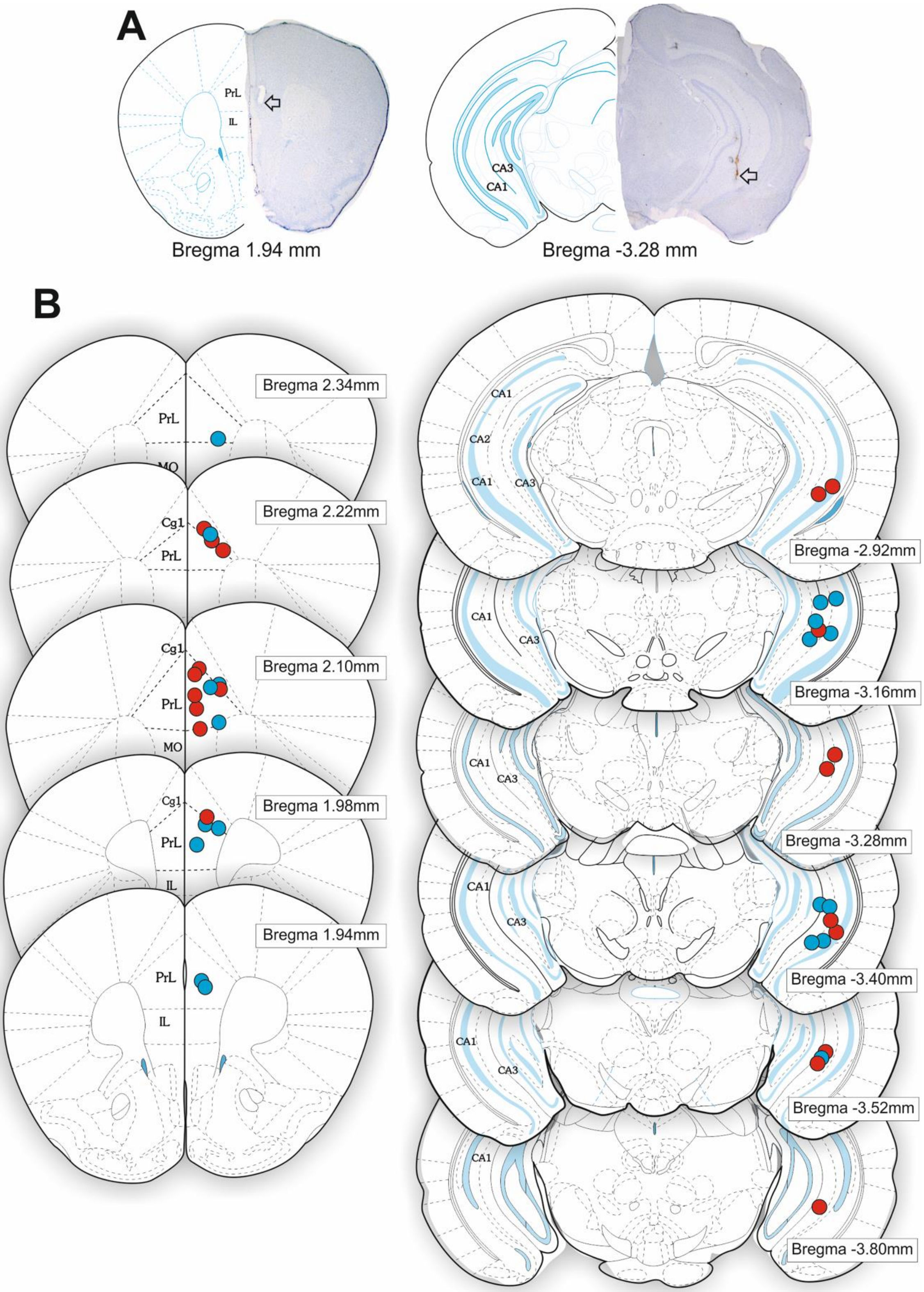

Figure S1. (**A**). Representative histological specimen (Nissl staining) showing electrode tracks from example animals, with arrows depicting estimated location of the recording electrode tip. (**B**). Histological verification of LFP recording electrode implantation sites in the mPFC and vHPC of all animals. Points indicate the position of electrode tips in Alloswitch-1 injected (red circles) and control (blue circles) groups on modified Atlas plates taken from The Mouse Brain Atlas [47]. Abbreviations: PrL – prelimbic cortex, IL – infralimbic cortex, Cg1 – cingulate cortex, MO – medial-orbital cortex, CA1-CA3 – field CA1-CA3 of the hippocampus.
